# Utilization of preconception care and associated factors among pregnant mothers in Fiche Town, Central Ethiopia: a community-based cross-sectional study 2021

**DOI:** 10.3389/fgwh.2023.1159693

**Published:** 2023-09-18

**Authors:** Negash Fetena, Abraham Negash, Alemi Kebede, Addisu Sertsu, Addisu Nega, Kabtamu Nigussie, Magarsa Lami, Elias Yadeta, Jerman Dereje, Aklilu Tamire, Fikadu Tolessa, Afework Tadele

**Affiliations:** ^1^ Yaya Gulale Woreda Health Office, North Shoa, Oromia, Ethiopia; ^2^ School of Nursing and Midwifery, College of Health and Medical Sciences, Haramaya University, Harar, Ethiopia; ^3^ Department of Population and Family Health, Institute of Health, Faculty of Health Sciences, Jimma University, Jimma, Ethiopia; ^4^ Department of Public Health and Emergency Management, Kellam Wallaga Zonal Health Office, Dembi Dolo, Oromia, Ethiopia; ^5^ School of Public Health, College of Health and Medical Sciences, Haramaya University, Harar, Ethiopia; ^6^ Department of Midwifery, College of Health Sciences, Salale University, Fitche, Ethiopia

**Keywords:** preconception, care, utilization, pregnant, Fiche, Ethiopia

## Abstract

**Introduction:**

Preconception care is an important preventive intervention for adverse pregnancy outcomes. It is recognized as a strategy to optimize women's health and pregnancy outcomes in Western countries. However, preconception care is underutilized in sub-Saharan Africa, like Ethiopia, where maternal mortality is high. Evidence is scarce in the study area about the prevalence and factors associated with preconception care utilization. Therefore this study aimed to assess the proportion of preconception care utilization and associated factors among pregnant mothers in Fiche town, central Ethiopia, 2021.

**Method:**

A community-based cross-sectional study was done from May 10 to June 25, 2021. A systematic random sample technique was used to choose 393 pregnant women for the study. A structured, pre-tested, interviewer-administered questionnaire was used to collect data. The data were entered into Epi Data version 3.1 and then exported into SPSS version 25 for analysis. A Bivariable and multivariable logistic regression analysis was used to check for the association. Odds ratio along with 95% was used to describe the association. Finally, a significant association was declared at a *p*-value less than 0.05.

**Results:**

388 respondents participated in this study, making the response rate 98.7%. Of total study participants only 84 (21.6%; 95% CI, 18, 25.8) utilized preconception care. The study found that diploma or higher level of education (AOR = 3.47, 95% CI: 1.27, 9.53), psychological and financial support from a partner (AOR = 3.86, 95% CI: 2.1, 7.10), joint discussion and plan with a partner (AOR = 3.32, 95% CI: 1.55, 7.13), history of chronic disease (AOR = 3.47, 95% CI: 1.67, 7.25), and good knowledge about preconception care (AOR = 2.42, 95% CI: 1.34, 4.38) were significantly associated with preconception care utilization.

**Conclusions:**

Overall, less than a quarter of the pregnant mothers utilized preconception care, indicating that awareness is very low. Pregnant mothers who have a higher educational level, have good communication and support from their partners, have chronic health problems, and have good knowledge about preconception care were more likely to utilize the service. Preconception care is a better opportunity to intervene and maintain the mother in the continuum of care.

## 1. Introduction

Preconception care (PCC) is the provision of biomedical, behavioral, and social health interventions to women and couples before conception to enhance their health and improve pregnancy outcomes (1–3). PCC is an important preventive health care intervention before conception for a couple (4, 5). It is also cost-effective in preventing adverse pregnancy outcomes, especially for those with chronic medical disorder (6–8).

Globally, 303,000 mothers die each year from maternal causes, with one in every 180 at risk; developing regions account for 99% of maternal deaths (9). About 66% of the global maternal mortality ratio (MMR) accounts for sub-Saharan Africa alone (10). In developing countries, maternal and neonatal mortality continues to be a serious public health problem (11, 12). To ensure and enhance maternal health and reduce MMR, preconception care is essential (11, 13).

Globally, less than one-third of women of reproductive age discuss their health status and its impact on pregnancy outcomes with a health professional (11, 13, 14). Moreover, the risks to reproductive health are still unacceptably high in many countries (14).

In Western societies, PCC is well recognized as a means of enhancing a healthy pregnancy and its outcome (14). Preconception care is widely recognized as being essential to ensuring the well-being of both women and their offspring (7, 15, 16). However, in most low-income countries, including Ethiopia, maternal health care may not begin until the pregnancy is well established or until more than half of the pregnancy has passed (11, 17).

Preconception care is a strategy for achieving sustainable development goal 3 (SDG 3): reducing maternal mortality to less than 70 per 100,000 live births and newborn mortality to as few as 12 per 1,000 live births by 2030 (18).

A health extension program was in place to address this problem and link the mother and newborn to the continuum of care, especially for those in remote areas (19).

Even though bad pregnancy outcomes, like malformation, are still a public health problem, the Ethiopian government has done its best to increase coverage and access to a continuum of maternity care (20, 21). The majority of policy initiatives have focused on enhancing women's health, increasing child survival, and reducing unfavorable pregnancy outcomes; however, there is an underuse of maternal preconception care (22, 23).

At the time women became aware of their pregnancy and started antenatal care (ANC) in Ethiopia, most of the fetal organs had developed (7, 24). PCC, which is the earliest link between maternal and newborn health, provides a window of opportunity to intervene accordingly and improve this gap (16, 25).

Preconception care is a key entry point to increase other services such as antenatal care, skilled delivery, and postnatal care and reduce adverse pregnancy outcomes (2, 26). Only, little is known and there are few studies on preconception care utilization in the study area. Therefore, this study was done to determine preconception care utilization and associated factors among pregnant women in Fiche Town, central Ethiopia, in 2021.

## 2. Method

### 2.1. Design, period and setting

A community-based, cross-sectional study was conducted from 10 May to 25 June, 2021, in Fiche Town, central Ethiopia. Fiche is a town located in the central part of Ethiopia, about 120 kilometers (km) from Addis Ababa. It is in the northern Shoa Zone of the Oromia region and has four kebele (lowest administrative unit in Ethiopia). The district has a total population of 104,345 people, according to the 2021 report obtained from the town health office, with 54,981 men and 49,364 women. Women in reproductive age groups in the town were 17,129. Data obtained from the health bureau of the Fiche town administration at the time of the study indicated the presence of about 936 pregnant women in the study area.

### 2.2. Source and study population

All pregnant women who lived in Fiche town for 6 months and above.

### 2.3. Sample size and sampling procedure

The single population proportion formula was used to calculate sample size with the following assumptions: Z /2 = 1.96, 95% confidence level, *p* = 18.2% of women use preconception care from a previous study conducted in northern Ethiopia (27), margin of error (d) = 4%, non-response rate = 10%. Finally, 393 people were chosen as the final sample size.

Data from the Fiche town health bureau, with the support of the health extension, was used to identify the number of pregnant women. Individual study participants were chosen using a systematic random sampling technique with *k* values of 2 (936/393 = 2.38). The first household to be included in the study was selected by the lottery method. If more than one pregnant mother was found in a single household, the lottery method was used to select study participants.

### 2.4. Variables of the study

Maternal utilization of preconception care is the study's dependent variable. Socio-demographic characteristics, obstetric and gynecologic characteristics, awareness and knowledge of preconception care, partner-related factors, and health system-related aspects are all independent variables.

### 2.5. Operational definitions

**Preconception care** is a comprehensive set of interventions that should be given to reproductive-age women before pregnancy to promote a healthy pregnancy and its outcome (28).

**Preconception care utilization**: Women will be considered to have used PCC if they received at least one of the following components of preconception care before pregnancy: counseling, disease screening and treatment, folic acid, vaccines, changing their diet, cessation of alcohol drinking, cessation of smoking, or creating a healthy environment (advice, treatment, and lifestyle modification**)** (26).

**Knowledge of women about PCC**: Eight knowledge questions were used to measure preconception care knowledge. Those who responded correctly 50% or above to preconception care knowledge questions were considered to have good knowledge, while those who scored less than 50% of correct responses were considered to have poor knowledge (29).

**History of adverse pregnancy outcome**: Previous pregnancy that ended in any of the following: preterm, low birth weight, abortion, stillbirth/intrauterine fetal death, birth defect (30).

### 2.6. Data collection instrument

A face-to-face interview was used to collect data using a pre-tested structured questionnaire. The tool consists of different parts that were developed from reviewing different literature and modified according to the local context (21, 31–33). Four bachelor's degree-holding nurses were used as data collectors and supervised by two MSC-holding nurses. During the data collection, regular supportive supervision and discussions with data collectors and supervisors were done. Onsite checking and review of the completed questionnaire were done by the principal investigator.

### 2.7. Data management and quality

Data collectors were trained for two days so that they became familiar with the aims of the study, its contents, sampling procedure, interviewing technique, data collection tools, and the issue of confidentiality. The questionnaire was first prepared in English and then translated to Afan Oromo and Amaharic by experts, then translated back to English by another person to ensure its consistency and accuracy. A pretest was carried out on 5% of the total sample size in one kebele, Sheraro town, which was placed outside of the main study area before the actual data collection. Following the pre-test, questionnaire modifications were made to improve the instrument's validity and reliability.

### 2.8. Data analysis

The editing, coding, and sorting of the collected questionnaire were done manually daily to check for completeness. After being checked for completeness, the data were entered into Epi-data Manager version 3.1 and then exported to SPSS version 25 for analysis. Descriptive statistics were done and the information was presented using tables, figures, and text.

There was no multicollinearity among the independent variables included in the model, and the maximum variance inflation factor was 1.058. The models' fitness was checked using the Hosmer and Lemeshow goodness of fit test. The internal validity of the tools was tested using Cronbach's alpha coefficient (0.76), which is good. Logistic regression was applied to analyze the association between dependent and independent variables. Bivariate analysis was employed to select candidate variables for multivariable analysis. Multivariable analysis was performed on variables with a *p*-value of less than or equal to 0.2. Multivariable analysis was carried out to assess the association between dependent and independent variables, and variables that have a *p*-value of less than 0.05 were identified as predictors. Adjusted odds ratios along with 95% confidence intervals were calculated for each of the independent variables in logistic regression to declare a significant association.

## 3. Results

### 3.1. Socio-demographic characteristics of pregnant mothers

A total of 388 pregnant women were interviewed, making the response rate 98.7%. The study participants' median age was 29 years, with an interquartile range of 24–33 years. More than half of the respondents were between 25 and 34 years old. Approximately half of the study participants (52.1%) have a monthly income of 5,000 ETB (91.68 USD) or less, while 229 (59%) were at the educational level of secondary school or above. The majority of the participants, 306 (78.9%), were married, and 81 (20.9%) of the women were merchants. Nearly half of the participants, 187 (48.2%), have a family size of 5 or above (Table 1).

**Table 1:** Socio-demographic characteristics of pregnant mothers living in Fiche town, 2021.

Variable	Category	Frequency	Percentage (%)
Age	15–24	99	25.5
	25–34	237	61.1
	35–49	52	13.4
The educational level of the pregnant mother	No formal education	75	19.3
	Primary education	84	21.6
	Secondary education	95	24.5
	Diploma and above	134	34.5
The educational level of the husband	No formal education	51	13.1
	Primary education	69	17.8
	Secondary education	131	33.8
	Diploma and above	137	35.3
Marital status	Single	21	5.4
	Married	306	78.9
	Divorced	27	7.0
	Widowed	12	3.1
	Separated	22	5.7
Number of family members	1–2	49	12.6
	3–4	152	39.2
	≥5	187	48.2
Average monthly income	<2,500 ETB	128	33.
	2,500–5,000 ETB	113	29.1
	>5,000 ETB	147	37.9
Occupation	Farmer	25	6.4
	Housewife	145	37.4
	Merchant	81	20.9
	Government employee	67	17.3
	Non-government employee	40	10.3
	Other	30	7.7
Occupation of husband	Farmer	32	8.2
	Merchant	46	37.6
	Government employed	106	27.3
	Non-government employed	76	19.6
	Daily laborer	28	7.2

#### 3.1.1. Obstetric history and reproductive health service-related factors

Almost two-thirds (64.7%) of study participants had 2–4 pregnancies, and 76.5% of study participants received financial and psychological support from a partner. Unplanned pregnancies accounted for 74 pregnancies (19.1%). Ninety-one (23.5%) had had previous miscarriages. We found that 62.4% of study participants had never heard of preconception care (Table 2).

**Table 2:** Obstetric history and reproductive health service-related factors among pregnant mothers living in Fiche town, 2021.

Variable	Category	Frequency	Percentage (%)
Gravidity	1	56	14.4
	2–4	251	64.7
	≥5	81	20.9
Parity	0–1	150	38.7
	2–4	213	54.9
	≥5	25	6.4
History of adverse pregnancy outcomes	Yes	91	23.5
	No	297	76.5
History of institutional delivery	Yes	265	68.3
	No	123	31.7
Had ever used family planning	Yes	260	67
	No	128	33
Status of pregnancy	Planned	314	80.9
	Unplanned	74	19.1
Joint discussion plan with partner	Yes	297	76.5
	No	91	23.5
Get financial and psychological support from a partner	Yes	112	28.9
	No	276	71.1
Heard about preconception care	Yes	146	37.6
	No	242	62.4
Screened for sexually transmitted disease	Yes	199	51.3
	No	189	48.7
Means of transport to reach a health facility	Foot	284	68%
	Public transport	91	23.5
	Private transport	33	8.5
Is the service delivery time convenient for you (at the health facility)?	Yes	352	90.7
	No	36	9.3
Does your partner enter the service delivery room with you?	Yes	53	13.7
	No	335	86.3
Having challenges to reach a health facility	Yes	152	39.2
	No	236	60.8
Preconception care utilization	Yes	84	21.6
	No	304	78.4
Presence of a chronic health problem	Yes	95	24.5
	No	293	75.5
Knowledge of preconception care	Good knowledge	106	27.3
	Poor knowledge	282	72.7

### 3.2. Utilization of preconception care

**Figure 1:**
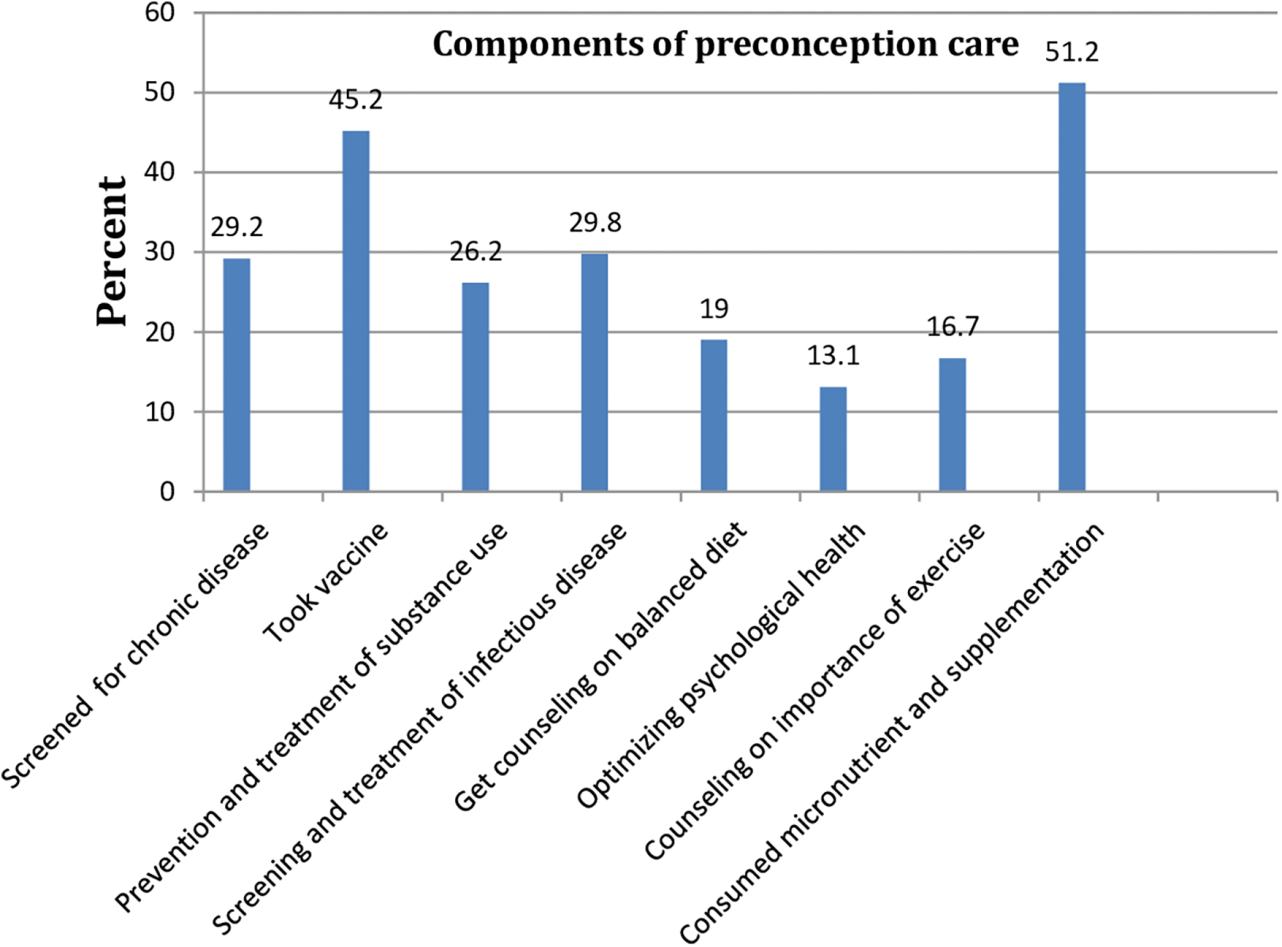
Proportion of World Health Organization components of preconception care utilization among pregnant women found in Fiche town, 2021.

Only 84 (21.6%; 95% CI: 18, 25.8) of the 388 study participants use at least one component of the World Health Organization's preconception care package. Micronutrient supplementation (i.e., iron, folic acid) is the most commonly used component of PCC while optimizing psychological health is the least used (Figure 1).

### 3.3. Factors associated with preconception care

Age, educational status, joint discussion and planning with a partner, getting financial and psychological support from a partner, partner inter ANC rooms, having a chronic health problem, having a bad obstetric history, hearing about preconception care, and having good knowledge about preconception care were associated with preconception care in binary logistic regression. However, in multivariable logistic regression, the educational status of the study participant, joint discussion and planning with the partner, getting financial and psychological support from the partner, having a history of chronic health problems, and knowledge of preconception care were significantly associated with preconception care.

Preconception care use was 3.47 (AOR = 3.47, 95% CI: 1.27, 9.53) times more likely among pregnant women who completed a diploma or a higher level of education. Preconception care utilization was 2.42 (AOR = 2.42; 95% CI: 1.34, 4.38) times more likely among pregnant mothers who had good knowledge (Table 3).

**Table 3:** A bivariable and multivariable logistic regression model of factors associated with preconception care among pregnant mothers in Fiche town, North Shoa, Ethiopia, in 2021.

Variable	Pre-conception care utilization		COR 95%CI	AOR 95%CI	*p*-value
	Yes	No			
Age					
15–24	16	83	1	1	
25–34	55	182	1.57 (0.85, 2.90)	0.91 (0.44, 1.88)	0.79
35–49	13	39	1.73 (0.76, 3.95)	1.67 (0.65, 4.33)	0.28
Educational status					
No formal education	6	69	1	1	
Primary education	15	69	2.5 (0.92, 6.82)	2.74 (0.92, 8.12)	0.069
Secondary education	20	75	3.07 (1.16, 8.08)	2.15 (0.74, 6.23)	0.15
Diploma and above	43	91	5.43 (2.18, 13.49)	**3.47 (1.27, 9.53)**	0.02
Joint discussion and plan with partner					
Yes	71	226	1.88 (0.98, 3.59)	**3.32 (1.55, 7.13)**	0.002
No	13	78	1	1	
Get financial and psychological support from her partner					
Yes	46	66	4.36 (2.62, 7.26)	**3.86 (2.10, 7.10)**	0.00
No	38	238	1	1	
Partner enters PCC service delivery room with his wife					
Yes	16	37	1.69 (0.89, 3.23)	0.45 (.018, 1.09)	0.07
No	68	267	1	1	
Had a history of chronic health problems					
Yes	42	53	4.73 (2.82, 7.97)	**3.47 (1.67, 7.25)**	0.001
No	42	251	1	1	
Had a history of negative pregnancy outcomes					
Yes	33	58	2.74 (1.63, 4.63)	1.03 (0.47, 2.25)	0.95
No	51	246	1	1	
Heard about preconception care					
Yes	46	100	2.47 (1.51, 4.04)	1.57 (0.84, 2.93)	0.16
No	38	204	1	1	
Knowledge of preconception care					
Good knowledge	35	71	2.34 (1.41, 3.90)	**2.42 (1.34, 4.38)**	0.003
Poor knowledge	49	233	1	1	

COR, Crude odd ratio; AOR, Adjusted odd ratio.

## 4. Discussion

In developing countries, like Ethiopia, where adverse maternal and neonatal outcomes are high, emphasizing preconception care is crucial. This community-based, cross-sectional study identified factors influencing PCC utilization among pregnant mothers in Fiche Town, central Ethiopia.

The findings of our study revealed that 21.6% (95% CI: 18, 25.8) of the respondents utilized preconception care. This finding was in line with studies done in the West Guji Zone (22.3%) (34), Mekelle (18.2%) (27), Hosanna Town (19%) (35), south-east Nigeria (23.4%) (36), and systematic review and meta-analysis in Africa (18.72%) (37).

Our study's findings, on the other hand, were lower than those of Mizan Aman (28.6%) (38), Southern Sri Lanka (27.2%) (39), Los Angeles 29.7 (40), Shanghai, China (42.2%) (41). Disparities in information accessibility, socioeconomic status, and the quality of the healthcare delivery system may all contribute to this variation (42).

This study's findings, however, are higher than those of Adet (9.6%) (26), Debre Birhan town (13.4%) (2), West Shoa zone (14.5%) (29), and Debre Tabor (15.8%) (43). The reason for this variation may be differences in the study population's level of education, culture, study setting, or year of the study. As the year elapses, there may be an increase in awareness and knowledge about the importance of preconception care, which leads to increased service utilization. This study finding is also higher than the systematic review and meta-analysis done by Ayele et al. (16.27%) (44). The possible justification for this may be a single study vs. systematic review and meta-analysis (SRMA). Our study was done at the community level while SRMA was a pooled result of both community and institutional-based studies.

Regarding factors, pregnant mothers who attended education to the level of a diploma or above were 3.47 times more likely to utilize PCC when compared to those who didn’t attend formal education. Studies done in Debre Birhan town (2), Adet (26), and China (45) support this finding. The reason for this may be that pregnant women with higher educational levels have more information and a better understanding of the importance of PCC, which is one of the driving factors for service utilization (34). Additionally, empowering women has a positive impact on maternity service utilization (46, 47).

Discussing and planning with a partner as well as obtaining psychological and financial support from her husband have a significant effect on PCC utilization. This finding was consistent with studies done in Mekelle (27), and Shanghai, China (45). This can be justified as, in developing countries, men are the chief decision-makers; therefore, their psychological and financial support enhances service utilization (31, 20).

The odds of PCC utilization were 3.47 times more likely among pregnant mothers who had a history of chronic health problems. This finding was supported by a study done in Mekelle (27). This may be because those with chronic health problems have followed up with and may have gotten information and advice from health professionals about the effect of their disease on pregnancy (48). They may also be advised on what to do before conception (49–51).

Knowing PCC increases service utilization by 2.42 times. This finding is supported by research conducted in the west Shoa zone (52), Mekelle (27), Mizan Aman (38), and Hosanna town(35). Evidence from Shanghai, China (45), also supports this study. This is an indicator that improving pregnant women's knowledge of the importance of PCC is an entry point for increasing service utilization (53, 54).

## 5. Strength and limitations

The study was conducted at the community level to address the pregnant mothers who did not visit the health facility. It is also the first study in the study area. However, the study was not without limitations, was cross-sectional, which did not indicate causation, and it was not multicenter. Additionally, there may be social desirability bias as it is interviewer administered.

## 6. Conclusion

This study indicated that the utilization of preconception care among study participants was found to be low. Reaching a high level of education, having a joint discussion and plan with a partner about pregnancy, getting psychological and financial support from a partner, and having good knowledge about preconception care were among the factors that enhanced service utilization. Having a chronic health problem is also one of the factors that promote service utilization. Involving partners in the maternity care continuum is critical to a positive maternal and neonatal outcome. Preconception is a better opportunity to intervene and maintain the mother in the continuum of care (55).

## Data Availability

The raw data supporting the conclusions of this article will be made available by the authors, without undue reservation.
